# Surgical treatment for common hepatic aneurysm. Original one-step technique

**DOI:** 10.1515/med-2020-0104

**Published:** 2020-09-11

**Authors:** Bruno Amato, Renato Patrone, Gennaro Quarto, Rita Compagna, Roberto Cirocchi, Georgi Popivanov, Vincenza Granata, Andrea Belli, Francesco Izzo

**Affiliations:** Department of Clinical Medicine and Surgery, University of Naples Federico II, Medical School, Naples, Italy; Department of Surgical Sciences, University of Perugia, Perugia, Italy; Department of Surgery, Military Medical Academy, Sofia, Bulgaria; Department of Radiology, “Istituto Nazionale Tumori IRCCS Fondazione G. Pascale – Napoli”, Naples, Italy; Department of Surgical Oncology, Hepatobiliary Unit, “Istituto Nazionale Tumori IRCCS Fondazione G. Pascale – Napoli”, Naples, Italy

**Keywords:** hepatic artery aneurysm, visceral aneurysm, aneurysm treatment, vascular stapler

## Abstract

**Introduction:**

Hepatic artery aneurysms are rare, and their treatment represents a challenge for the surgeons.

**Materials and methods:**

A new technique is presented for common hepatic artery (CHA) aneurysm: it requires minimal vascular surgical dissection and only one linear vascular stapler is applied at the bottom of aneurysm. Aneurysm exclusion is easily obtained, which allowed retrograde thrombosis. Liver blood supply is ensured to the right and left hepatic artery, through the gastroduodenal artery, and can be previously monitored, with temporary clamping of the section area, by visual control, enzyme evaluation and intraoperative ultrasound examination. We reported an open surgical treatment, with simultaneous removal of hepatic and adrenal metastases, secondary to colon cancer.

**Results:**

The duration of vascular surgery was 30 min and did not involve complications. Postoperative controls confirmed the efficacy of the procedure.

**Discussion:**

This original technique can be added to the various open and endovascular techniques so far described for the treatment of a CHA aneurysm. It is advisable as open surgery, mostly in case of associated pathologies.

**Conclusions:**

The authors believe that this “one shot” technique by vascular staple of the distal part of CHA is minimally invasive and effective to obtain the exclusion of the aneurysm.

## 1 Introduction

The hepatic artery aneurysm has a significant incidence (20–25%) in the area of visceral artery aneurysms, but a low incidence compared with abdominal aortic aneurysms (0.2–1.0%) [1,2]. Their etiology may be atherosclerotic (about 30% of cases, especially in elderly patients), but other causes have also been described, such as for other visceral aneurysms, related to vasculitis, fibromuscular dysplasia, trauma, iatrogenic causes or infectious [3,4,5,6,7]. The first series reported in the literature dates back to 1908 (Rolland) [8]: it describes 40 cases of hepatic artery aneurysms, mainly attributable to infectious origins, mostly in young patients. The advent of ultrasound, for both the diagnosis of abdominal pathologies and the control of pregnant women, has contributed to the early identification of aneurysmal dilations of the hepatic artery in an increasing number of cases [9,10,11].

Common hepatic artery (CHA) is affected in 67% of cases of hepatic district aneurysm, mainly without any symptoms, but sometimes show jaundice or clinical signs similar to biliary colic [12]; in 32% of cases, the localization or extension of the aneurysm to gastroduodenal artery (GDA) was observed [1].

Treatment of CHA aneurysm is considered an infrequent occurrence in both urgency and election [13,14]. Indications for the treatment of a hepatic aneurysm derive from being symptomatic (by compression of the biliary tract), from causing distal embolisms towards the liver or from risk of rupture, when its dimensions show a rapid growth or exceed 2 cm of transverse diameter [15,16,17]. Emergency treatment of CHA aneurysms, burdened by a high index of complications, is instead foreseen in the event of aneurysm fissuring and for the control of bleeding, related to its rupture: This complication occurs in 70–80% of the observed cases when the dimensions exceed 3 cm of transverse diameter [16,17].

Surgical treatments proposed so far are manifold and include:

– resection of aneurysmal sac and direct suturing of the two ends of the vessel,
– interposition of a prosthetic segment of saphenous vein or synthetic vascular graft,
– aneurysmorrhaphy and
– only proximal and distal ligation of aneurysmal sac.

The different techniques testify to the difficulties in accessing the celiac aorta, which is not usual for most surgeons, in addition to the opportunity to guarantee adequate blood supply to the liver after surgery.

The overwhelming advent of endovascular procedures has so far not defined the best treatment even if actual indications are increasingly oriented toward endovascular techniques, by application of stents or endoprosthesis to protect the integrity of the vessel wall or by application of “coils” to induce occlusion of aneurysm sac, when vascular supply to the liver can be guaranteed by the GDA [17,18,19,20].

Open surgery still find indications mostly in case of the associated abdominal diseases: in that case, the treatment of the hepatic aneurysm can however determine a considerable lengthening of the operation time, the need for extensive vascular dissections and the use of prosthetic materials, with the risks of thrombotic, hemorrhagic or infectious complications [21,22,23,24].

In the literature, there have been, till now, no reports of surgical treatment for hepatic artery aneurysm associated with the supplementary abdominal surgery.

We here report a case of surgical treatment of hepatic artery aneurysm associated with the treatment of multiple metastases from the colon cancer. Vascular technique adopted in this case had the objective of minimizing operation time and the use of prosthetic materials: in fact, it was obtained through the exclusion of the CHA aneurysm using a “retrograde thrombosis technique” of aneurysmal sac, obtained by applying a single vascular linear mechanical stapler placed at the bottom of aneurysmal sac, tangentially to the GDA pathway, avoiding in this way total exclusion of the blood supply to the liver.

## 2 Materials and methods

The technique is reported on the experiment performed on a patient: a woman aged 76 year-old (T.M.). She presented history of associated HbsAg+ liver disease, cholecystectomy and fusiform aneurysm of basilar artery. In August 2108, she was diagnosed with carcinoma of the sigmoid rectum, and in September of the same year, she underwent left video-laparoscopic hemicolectomy at the Abdominal Oncological Surgery Unit of “Pascale” IRCCS in Naples, Italy. Primitive surgery was free of major complications, and her histology reports were as follows: adenocarcinoma pT4b, pN2a, pV1, pR0 and G2.

**Figure 1:**
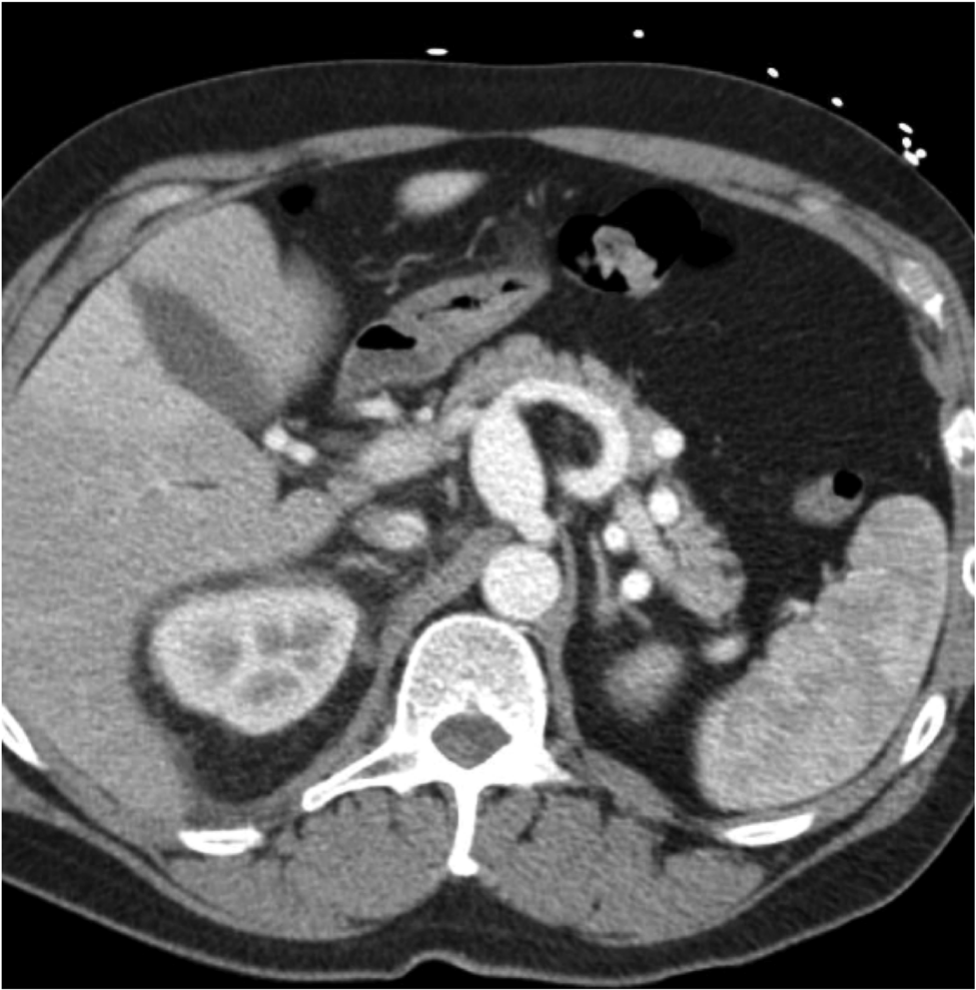
Preoperative CT scan of hepatic artery aneurysm (CHA).

During her clinical follow-up in July, the patient performed CT abdomen, which showed, in sixth hepatic segment, a metastatic lesion of 32 mm in diameter, associated with aneurysmal dilatation of CHA, partially thrombosed. Therefore, she underwent chemotherapy (FOLFOX scheme) from March 29, 2019, to August 26, 2019. Subsequently she performed CT scan: it showed a liver mass of the 6th segment (30 mm × 24 mm) and an aneurysmal dilatation of the CHA (34 mm × 42 mm) (Figure 1), without the involvement of confluent arterial vessels. GDA, right hepatic artery (RHA) and left hepatic artery (LHA) size were normal indeed. There was a hypodense mass (63 mm × 50 mm), probably metastatic, on the left adrenal gland.

On September 7, 2019, the patient underwent open surgery for liver resection, left adrenalectomy, CHA aneurysm treatment and splenectomy.

In preoperative evaluation, she underwent spirometry tests (25% FEV1), followed by a chest computer tomography (CT) scan (free from nodular lesions) and brain control CT for basilar artery aneurysm (resulted stable). Patient’s blood values were normal, and preoperative assessment classified her in ASA III risk class, which requested the availability of postoperative intensive care unit, as usual for major multiple oncological resections.

Patient was widely informed about possible complications and was provided with all information necessary to understand the suggested intervention and patient’s risks. Written informed consent was obtained from the parents, and an institutional review board approved this pilot plan of treatment.

General anesthesia was applied as usual. Bi-subcostal incision was performed, and the operative field was exposed by Balfour and Holzbach retractor for liver surgery.

**Figure 2:**
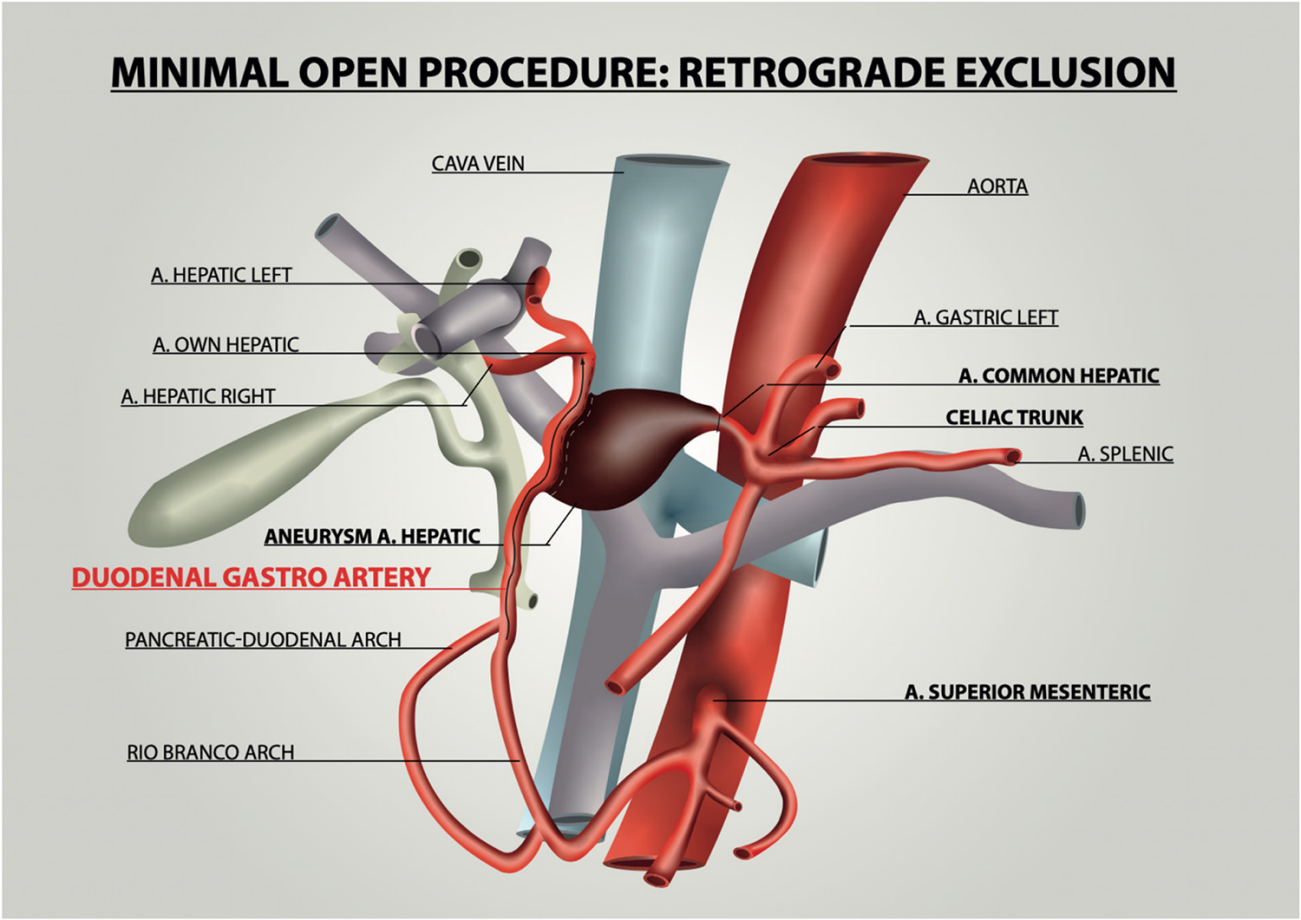
Anatomical representation of CHA aneurism treatment by exclusion by the section of bottom of aneurysm and retrograde thrombosis, ensuring liver blood supply from GDA.

First surgical step was the isolation of distal segment of CHA aneurysm and of GDA, RHA and LHA vessels. Second operative step involved temporary clamping (without heparin administration) of distal CHA, tangentially to the GDA pathway, to maintain GDA patency and patient’s capability to provide blood supply for RHA and LHA (Figure 2). This second surgical step was necessary to control adequate liver supply. The duration of these two surgical steps was approximately 20 min.

Third and fourth surgical steps has been therefore aimed at carrying out a radical removal of the left adrenal gland and a wedge resection of the VI hepatic segment. The duration of the third and fourth surgical stages required about 80 min: during these second two phases, distal CHA clamping was maintained.

During the fourth phase, hepatic function was verified by liver function tests: the liver transaminases aspartate transaminase (AST or SGOT) and alanine transaminase (ALT or SGPT) are useful and early biomarkers of liver ischemia, which is evidenced by a threefold increase from baseline after 30 min of significant liver ischemia [25].

The results of these analyzes showed nonsignificant increase in LDH values (8% increase compared to preoperative values: from 331 to 359) and equally nonsignificant increase in the GOT values (18% increase: from 19 to 23 and in the GPT values (increase of 50%: from 11 to 18; Table 1).

**Table 1:** Intraoperative blood enzymes

	Preclamping	Postclamping (20 min)	% variation
LDH	331.4	359.6	+8.2
GOT	19.6	23.4	+18.6
GPT	11.7	18.3	+46.3

An intraoperative analysis of flow parameters by ultrasound (10 MHz probe) at GDA, RHA and LHA levels was also conducted by the vascular team.

The examination of liver enzymes, together with echo-doppler blood velocity examination of RHA and LHA and visual inspection of liver surface (unchanged during the whole period of CHA clamping, in comparison with the previous free operative phase), confirmed adequate liver blood supply during CHA clamping and safely permitted the subsequent application of a linear vascular stapler (Echelon-type, vascular) to carry out hemostatic suture of the end of CHA, tangential to the GDA, and patient’s following section. Alternatively, surgery for the treatment of CHA aneurysm would have involved the implantation of a prosthetic or venous graft between the abdominal aorta and the bifurcation of the CHA: such surgery would have provided much more prolonged surgical times and surgical difficulties.

**Figure 3:**
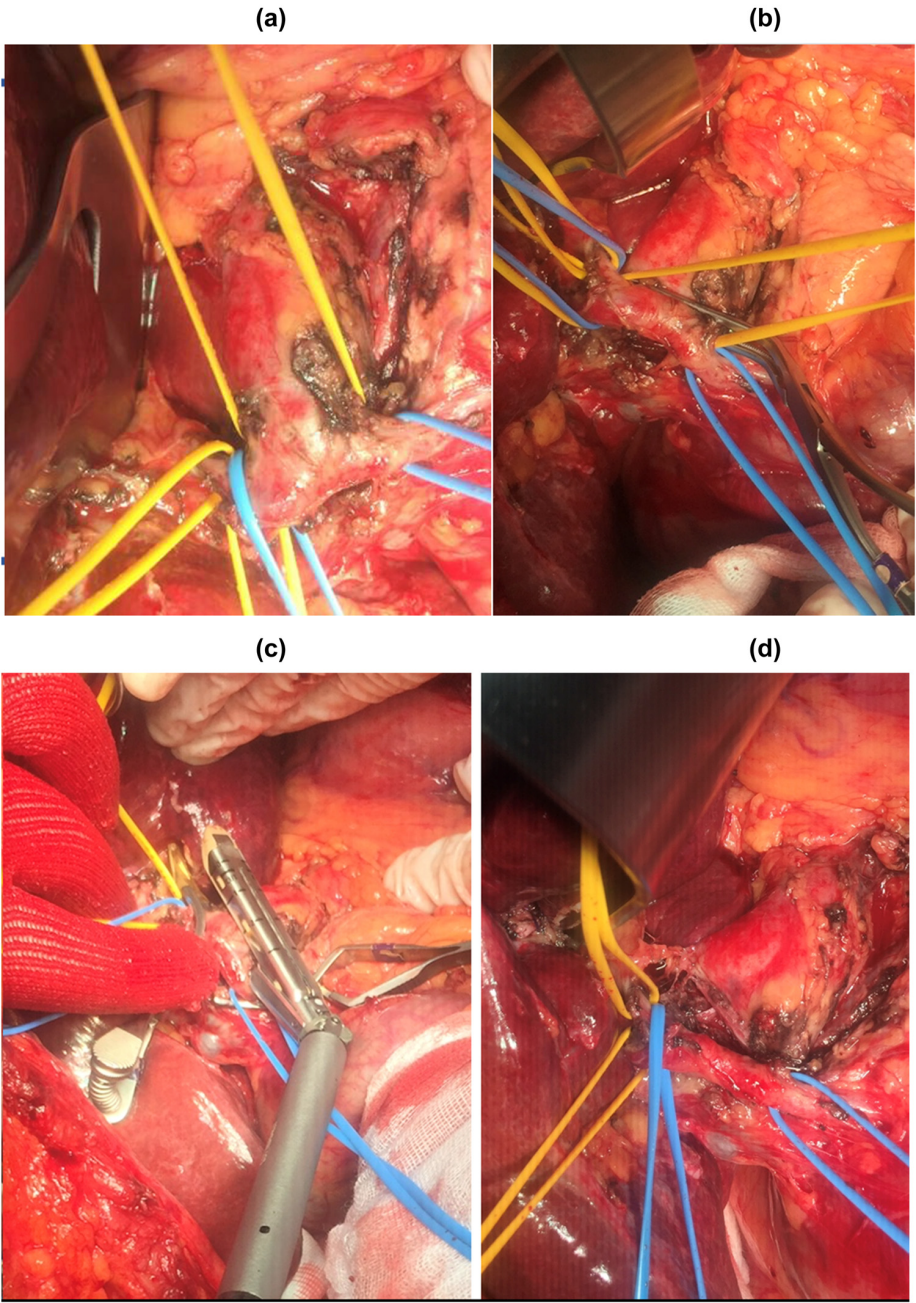
Intraoperative time: (a) isolation of distal segment of CHA aneurysm and of GDA, RHA and LHA vessels; (b) temporary clamping; (c) Stapler (Echelon-type, vascular) was applied at the end of the CHA, tangentially the lateral wall of the GDA, determining a cul de sac of CHA aneurysm and creating the lateral wall of the GDA, which continues into the bifurcation of the RHA and LHA; (d) CHA aneurysm sac was excluded from circulation. GDA provides blood supply for RHA and LHA

Stapler was applied at the end of the CHA, tangentially the lateral wall of the GDA, determining a cul de sac of CHA aneurysm and creating the lateral wall of the GDA, which continues into the bifurcation of the RHA and LHA (Figure 3). In this way, CHA aneurysm sac was excluded from circulation, going in short time to a retrograde thrombosis up to the emergence of the left gastric artery (LGA) and of the splenic artery (SpA): patency of these two vessels was then confirmed by ultrasound intraoperative control.

15 min were required for ultrasound intraoperative analysis and the application of the stapler. This last surgical step (suture and section of the CHA and subsequent verification of liver integrity) concluded the main operative vascular phases of the operation. Then, an accurate control of residual bleeding was carried out, and appropriate drainages were positioned.

## 3 Results

The overall duration of the surgery was 150 min. The patient was admitted for 3 days in the postoperative intensive care unit, where she underwent blood tests (day 1, 2 and 3), which confirmed the stable liver function (by enzyme parameters: LDH, GOT and GPT) and the stability of other serological parameters (Table 2).

**Table 2:** Post-operative blood enzymes

	First post-op day	Second post-op day	Third post-op day
Hemoglobin	9.8	10.1	9.6
WBC	6.2	6.8	7.3
Albumin	4.8	4.2	4.3
LDH	382.2	364.1	376.0
GOT	26.3	22.6	24.1
GPT	20.5	19.9	20.7

Patient resumed oral feeding and mobilization on day 3. On day 5 post-op, she presented with febrile symptoms (38.5°C), and therefore, antibiotic therapy with ceftriaxone was started until her discharge on the tenth day after surgery.

**Figure 4:**
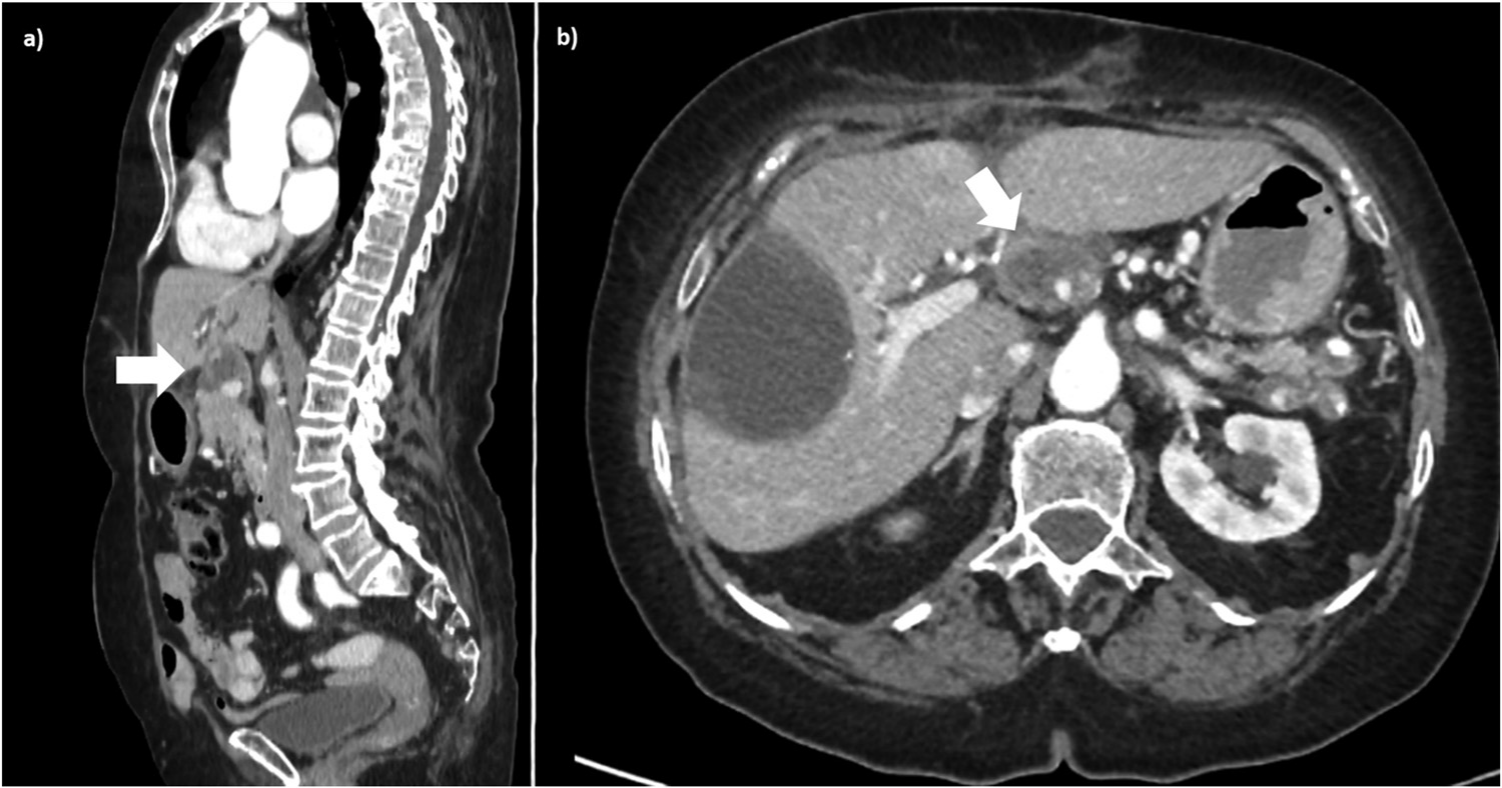
6 months postoperative follow-up. CT scan during arterial phase of the contrast study, in MPR sagittal plan (a) and in axial plane (b): the arrow shows complete thrombosed CHA aneurysm and his vascular exclusion.

Before her discharge, the patient underwent angio-CT scan, which confirmed CHA aneurysm thrombosed and excluded. Blood supply of the liver appeared normal. Ultrasound investigation carried out before discharge showed regular blood flow in RHA and LHA, celiac tripod (CT), LGA and in SpA and also showed complete thrombosis of CHA aneurysm sac (Figure 4).

Angio-CT scan control, performed for oncological follow-up after 6 and 12 months from last surgery, confirmed the absence of further metastatic foci, and the same vascular theatre was found on ultrasound examination at the patient’s discharge.

## 4 Discussion

Decision to carry out the associated treatment of liver and adrenal metastases with CHA aneurysm in a single surgical procedure came from a common evaluation and agreement among the oncology, abdominal surgery and vascular surgery teams. The choice of associated treatment of hepatic and adrenal metastases and also of CHA anerysm arose from the aim to obtain a radical cancer treatment and not to neglect the aneurysmal pathology, due to its considerable size and its rapid growth: these factors have made the risk of early aneurysm rupture very high indeed [16,17]. The option to carry out a preliminary endovascular procedure with the application of a stent or endoprosthesis was excluded due to the difficulties connected to the tortuosity of the vessel and the need to use subsequent antiaggregation therapies considered incompatible, due to bleeding risk, compared to the subsequent removal of the metastatic hepatic and adrenal masses. Similarly, the hypothesis of performing an endovascular exclusion with metal coils, or other embolization techniques, was not adopted for the risk of ischemia of the hepatic parenchyma, which this technique exhibits [25,26,27].

Although we report that the current guidelines indicate preferable endovascular procedures over open surgery, as they are less invasive and short lived [28,29], the authors believe that the indication of the most appropriate treatment for CHA aneurysms is linked to multiple factors such as the site and the anatomy of the aneurysm, its dimensions, the etiology, and the risk of rupture, also general condition and life expectancy of each patient, the experiences of vascular surgical and radiological team and all the vascular and oncological therapies necessary or foreseeable in the short term [30,31,32].

The choice of performing the surgical treatment by “retrograde thrombosis” herein described, with accurate control of liver blood supply through the application of a single linear vascular stapler, which has been appropriate, considering the limited surgical time required and to the result obtained, with the full guarantee of avoiding risks of aneurysmal rupture and damage to liver vascularization. At the same time, the patient was subjected to a single operative procedure, which is low cost and consumes less time (in comparison with a preliminary endovascular treatment for the hepatic aneurysm), without radiation, contrast agents and auxiliary drugs to endovascular treatment. In addition, the presented technique in this specific patient has particular clinical characteristics for its associated pathologies, which have conditioned the surgical choices, imposing a surgical program as limited in its duration, but at the same time capable of identifying, although it is a single case, the quality of the applied technique. For this reason and for the rarity of aneurysmal pathology found in the CHA, it would be difficult to collect a larger number of cases.

## 5 Conclusions

In the presented case (also considering the good result obtained, free of complications), we believe to have described an effective new therapeutic option for a rapid and minimally invasive surgical treatment of CHA aneurysm, using an original technique, so far not yet described in the literature. The described technique is also practicable not only in the case of surgical interventions associated with other pathologies but also as an isolated surgical procedure for CHA aneurysm, in conditions similar to those case presented (related to the size and the rapid growing of the aneurysm), thanks to the speed of execution and for the possibility to perform a preventive control of the hepatic blood supply. Finally, the authors believe that this proposed technique can also be performed through video laparoscopy, with indications and limits, which further study can identify.
